# Analog Resistive Switching Devices for Training Deep Neural Networks with the Novel Tiki-Taka Algorithm

**DOI:** 10.1021/acs.nanolett.3c03697

**Published:** 2024-01-11

**Authors:** Tommaso Stecconi, Valeria Bragaglia, Malte J. Rasch, Fabio Carta, Folkert Horst, Donato F. Falcone, Sofieke C. ten Kate, Nanbo Gong, Takashi Ando, Antonis Olziersky, Bert Offrein

**Affiliations:** † IBM Research Europe - Zürich, Rüschlikon, Zürich CH 8803, Switzerland; ‡ IBM Research - Yorktown Heights, Yorktown Heights, New York 10598, United States

**Keywords:** analogue, RRAM, Tiki-Taka, training

## Abstract

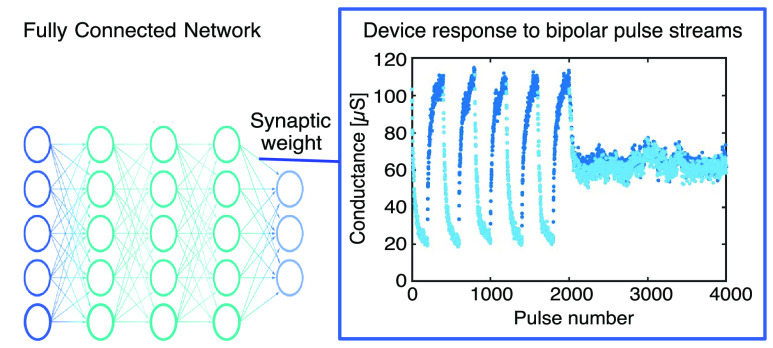

A critical bottleneck for the training of large neural networks (NNs) is communication with off-chip memory. A promising mitigation effort consists of integrating crossbar arrays of analogue memories in the Back-End-Of-Line, to store the NN parameters and efficiently perform the required synaptic operations. The “*Tiki-Taka*” algorithm was developed to facilitate NN training in the presence of device nonidealities. However, so far, a resistive switching device exhibiting all the fundamental Tiki-Taka requirements, which are many programmable states, a centered *symmetry point*, and low programming noise, was not yet demonstrated. Here, a complementary metal-oxide semiconductor (CMOS)-compatible resistive random access memory (RRAM), showing more than 30 programmable states with low noise and a symmetry point with only 5% skew from the center, is presented for the first time. These results enable generalization of Tiki-Taka training from small fully connected networks to larger long-/short-term-memory types of NN.

The compute effort required for training neural networks is growing fast, doubling every three to four months,^1^ outpacing Moore’s scaling law.^2^ To address this challenge, computer architectures are being reshaped to tackle their main sources of inefficiency such as low memory bandwidth and poorly distributed workloads. Modern AI accelerators based on digital electronics enhance computational parallelism and improve energy efficiency by bringing memory closer to the processing unit.^3^ However, the on-chip memory capacity is limited and cannot accommodate the full size of today’s large NN parameters. Therefore, communication with off-chip memory is required, representing the main source of computational inefficiency.^4^

To overcome this memory wall, a novel hardware architecture, consisting of crossbar arrays of nonvolatile resistive switching devices, has been proposed.^5,6^ It was shown that calculating the interconnections between neurons represents the largest computational effort for NN inference, accounting for more than 98% of the required operations.^7^ Using crossbar arrays, the same calculations are performed directly in-memory,^5,6^ and their complexity of execution is reduced to O(1).^8^ Moreover, by implementing parallel updates of the array conductances, the NN learning rule can be performed more efficiently and faster than in digital AI accelerators.^9^ Since the conventional training algorithms demand linear and symmetric synaptic updates, while common resistive-switching devices exhibit nonideal characteristics,^10,11^ a technological challenge exists to improve the device properties.

“*Tiki-Taka*”^12^ was recently developed to mitigate such issues. It is algorithmically similar to momentum stochastic gradient descent (SGD)^13^ but optimized for analogue resistive hardware. It comprises two phases, gradient accumulation and weight updates, and employs two crossbar arrays. One crossbar array maps the accumulated gradients (matrix A) into its array conductance (*G*) values. The other crossbar array maps the NN weights (matrix C). In the first phase, the gradients are accumulated in a leaky fashion to generate momentum. Then, this information is transferred, updating matrix C. Before starting the gradient accumulation phase, the devices mapping matrix A must be prepared in a state where the *G* changes upward and downward are equal, called the “*symmetry point*”.^12^ Around this state, the symmetry conditions are sufficient to compute the gradients with the correct sign.^12^ Hence, the fundamental benefit of Tiki-Taka is to confine the symmetry requirements, from the full *G* window, to a local symmetry point.

The Tiki-Taka algorithm relies on two essential requirements. First, the device must show analogue resistive switching in both directions, so that a symmetry point exists within its *G* window. Second, the *G* updates must be driven by a stream of pulses of identical amplitude and duration. Whenever two pulses of opposite polarity coincide at the terminals of one device, they yield a sufficiently high voltage, generating a *G* update.^9^ With such an implementation of the learning rule, all of the array elements are updated in parallel, accelerating the execution of the training task.

In previous works, the weight update was performed row by row^14^ or device by device.^15^ In our recent work,^16^ NN training with parallel crossbar updates was demonstrated. We implemented artificial synapses using resistive random access memory (RRAM) devices. Compared with other memristor technologies, such as Ferroic tunnel junctions (FTJs), RRAM devices offer two main advantages. First, they exhibit analogue long-term potentiation and depression characteristics using identical input voltage pulses, while FTJ structures require incremental pulse amplitudes.^17^ Second, the RRAM memory cell is highly scalable because of the filamentary nature of its resistive switching mechanism. Compared to the electrochemical-RAM (ECRAM) structures,^18,19^ the filamentary RRAM devices show faster programming and require voltage pulses of lower amplitude. Compared to volatile charge-based synaptic structures,^20^ the filamentary RRAM devices enable denser array implementations. Moreover, the programmed analogue conductance states show longer retention times. Hence, RRAM devices are worthwhile investigating for the training of complex data sets, requiring long learning times.

In our recent work,^16^ we also showed that the Tiki-Taka algorithm improves the training accuracy compared to the SGD method. We could perform the training of a fully connected network (FCN) on a reduced Modified National Institute of Standards and Technology (MNIST) data set, but devices with enhanced properties are required for scaling to larger networks. In a recent collaboration,^21^ we showed that the property *number of states* is improved upon applying short programming pulses (down to 300 ps).

Compared to such previous literature, in this work we present devices combining improvements in every important property for the Tiki-Taka algorithm, such as a higher number of states^16^ and a more centered symmetry point.^21^ Moreover, we show a stable symmetry point over cycling, and we demonstrate the robustness of the algorithm against device-to-device variability. The favorable device properties are conserved even after millions of programming pulses.

To achieve these excellent results, we further optimized the material stack presented in our previous study,^22^ based on the TiN/conductive-TaO_*x*_/HfO_2_/TiN structure. We engineered the HfO_*x*_ deposition conditions to reduce the operational voltages and currents, facilitating a future integration of advanced CMOS technology. Moreover, the process flow was modified to scale down the unit cell area, toward a high density of memory integration in the Back-End-Of-Line (BEOL). We modeled the device response and performed NN training simulations using an optimized version of the Tiki-Taka algorithm (*TTv2*^23,24^).

A structural representation of the RRAM devices discussed in this work is depicted in Figure 1(a). The device active layers are the conductive-TaO_*x*_ and the HfO_*x*_, providing the analogue bidirectional switching properties discussed in our previous work.^22^ Other details on the material properties are reported in the Supporting Information Methods section.

**Figure 1:**
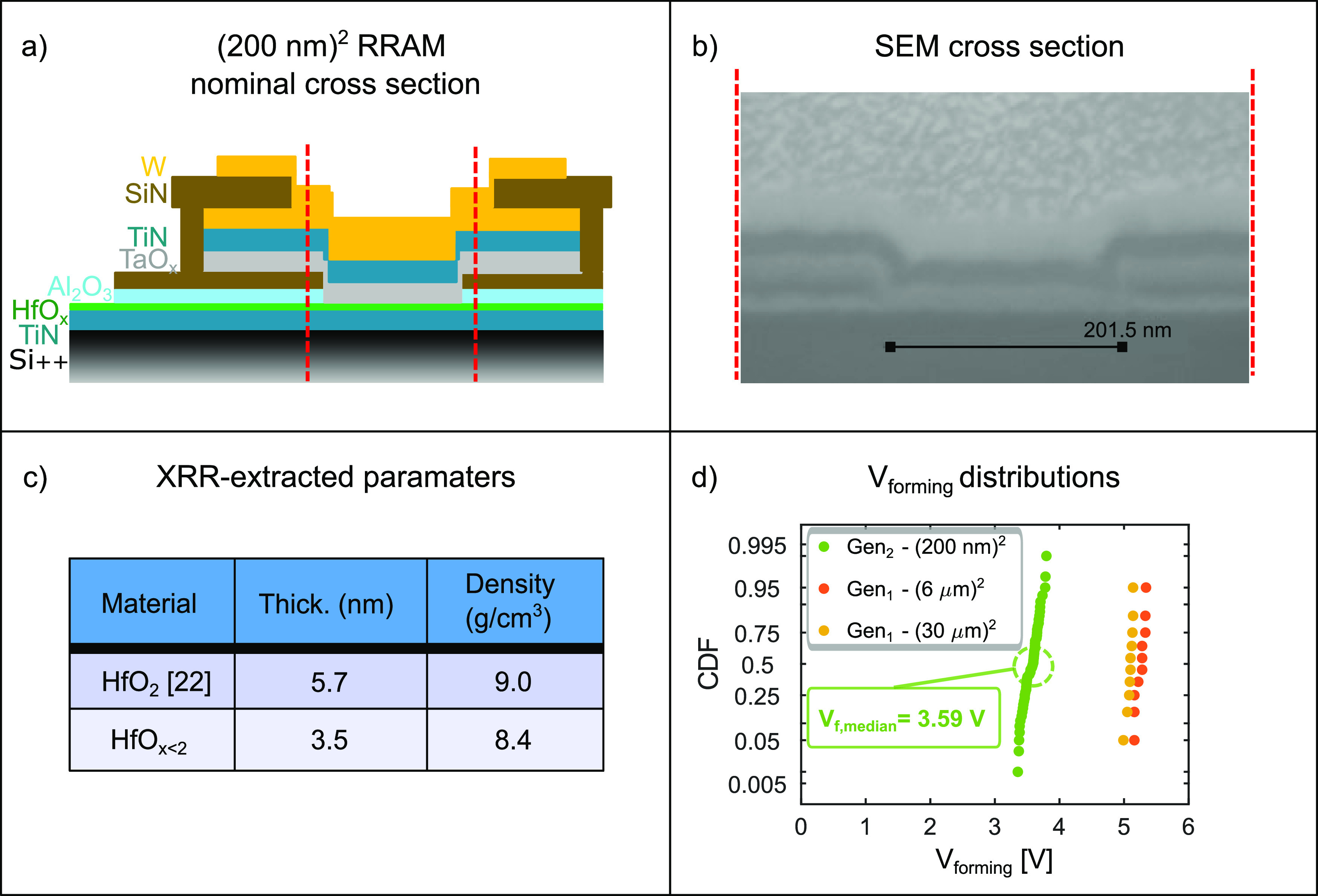
(a) Sketch of the RRAM cross-section profile. (b) SEM cross-sectional image of a (200 nm)^2^ Gen_2_-RRAM. (c) Comparison between the thickness and density of the HfO_2_ and the HfO_*x*<2_ thin films used in Gen_1_- and Gen_2_-RRAMs, respectively. (d) Cumulative distributions of the forming voltages from Gen_1_ and Gen_2_ devices, in orange and green, respectively. Various device areas are compared.

To compare the former^22^ and the new batch of RRAM devices, we refer to *Generation 1* (Gen_1_) and *Generation 2* (Gen_2_). Gen_1_-RRAMs were fabricated using a “*mesa*” approach, where a dry process is used to etch the top W, TiN, and TaO_*x*_ layers sequentially. These materials showed different etching selectivities, leading to isotropic patterning. For this reason, the scalability of the unit cell area below sub-μm nodes turned out to be challenging (see Supporting Information Figure S1). Therefore, we modified the process flow for the new Gen_2_ devices. In the new approach, the active area is defined by etching a “via” through the SiN/Al_2_O_3_ bilayer, as illustrated by the cross-section sketch in Figure 1(a).

Atomic force microscopy measurements reported in Supporting Information Figure S2 confirmed that this process based on wet etching does not harm the HfO_*x*_ layer. Scanning electron microscopy (SEM) was used to image the cross-section of a (200 nm)^2^ RRAM device, as depicted in Figure 1(b), showing accurate control of the unit cell area.

High filament forming voltages (more than 5 V) were measured in Gen_1_ devices.^22^ To decrease the forming voltages, we modified the plasma-enhanced atomic layer deposition (PE-ALD) of the HfO_*x*_ layer. The goal was to reduce the density and the thickness of the deposited thin film and to increase the leakage current density, hence lowering the forming voltages.^25−27^ The O_2_ plasma time was reduced from 10 to 1 s, which led to a reduction of the film density, from 9 to 8.4 g/cm^3^, as measured by X-ray reflectivity (XRR) characterization (see Figure 1(c)). Moreover, the number of ALD cycles was reduced from 50 to 34, and the film thickness decreased from 5.7 to 3.5 nm, as confirmed by the XRR measurement (see Figure 1(c)).

In the Gen_1_ chip, we observed that devices with smaller areas exhibit slightly higher forming voltages.^22^ This can be explained by considering the filament forming as a stochastic process, involving percolative paths across the metal-oxide stack.^28^ Gen_2_ devices are ∼1000 times smaller, but they show much lower forming voltages (around 30%), as shown in Figure 1(d). We attribute this result to modified HfO_*x*_ properties.

The described process flow and materials are CMOS- and foundry-compatible.

The procedure designed for analogue programming is described in Figure 2(a). We apply trains of identical voltage pulses but alternating polarity in batches of 200. We repeat this scheme multiple times, to evaluate the stability of the exhibited *G* window. Finally, we reduce the batch size to 1, hence alternating a pulse up to a pulse down, to shift the *G* states toward a symmetry point, where up and down resistance changes are equal.^12^ This procedure simulates the programming conditions imposed during a Tiki-Taka training.

**Figure 2:**
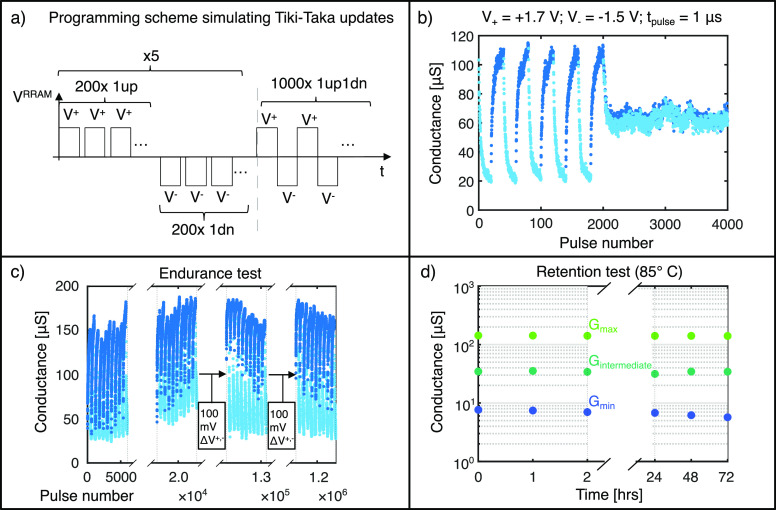
(a) Electrical test scheme used to assess the analogue switching properties for the Tiki-Taka algorithm. (b) Gen_2_ device bidirectional accumulative response and symmetry point. The synaptic depression and potentiation are colored light blue and dark blue, respectively. The applied waveforms have amplitudes of +1.7 V and −1.5 V for the down and up directions, respectively, and a duration of 1 μs. (c) Endurance test for the analogue *G* swing. To keep a stable window, we increase the pulse amplitudes by Δ*V* = 100 mV after 10^5^ and 10^6^ pulses. (d) Retention of three analogue states (*G*_min_, *G*_max_, and *G*_intermediate_) at 85 °C up to 72 h.

A typical device response is shown in Figure 2(b). A stable *G* window with many analogue states is found, followed by a centered symmetry point. Compared to our previous work,^22^ Gen_2_ devices show a reduction of the *G*_min_ and *G*_max_ values by a factor ∼2.5×, improving the power efficiency of the multiply accumulated operation.

We tested the endurance of the analogue *G* window by alternating positive and negative batches up to more than 10^7^ pulses. The entire data collection from this test is reported in Supporting Information Figure S3, while in Figure 2(c) we show the main results.

After around ∼10^5^ pulses, the *G*_max_ state drifts downward, as shown in Supporting Information Figure S3(d). Nonetheless, the original *G* window can be restored by increasing the pulse amplitudes (*V*^+^ and *V*^–^) by 100 mV, as shown in Figure 2(c).

Over the next 10^6^ pulses the *G*_max_ value drifts downward again (see Supporting Information Figure S3(f)). Once more, after increasing the programming voltages by 100 mV, the initial *G* window was recovered, as reported in Figure 2(c). The correction of the pulse amplitudes helps stabilize the symmetry point after repeated programming. In Supporting Information Figure S9, we show a stable symmetry point even after 10^6^ programming pulses thanks to the pulse amplitude correction. Moreover, in the same Supporting Information Figure S9, we show that the cycle-to-cycle variability of the symmetry point is below 10%. We also show in Supporting Information Figure S3 that the device exhibits analogue potentiation and depression after more than 10^7^ pulses, but the *G* window has now strongly widened compared to the initial one (see Supporting Information Figure S3(h)).

Since we target NN training applications, where weight updates occur frequently, the retention of the analogue states is studied for up to 72 h. The results are depicted in Figure 2(d). Generally, excellent stability is observed, except for a minor skew of the *G*_min_ state (downward drift of 4% after baking for 72 h at 85 °C). Our devices show long-term stability of the conductive states, with time scales comparable to other high performance memristor devices.^29^ Programming of up to 32 states and their temporal stability are displayed in Supporting Information Figure S10.

We introduce new figures of merit for the Tiki-Taka algorithm, combining the properties of the full *G* swing and the symmetry point (SP).

The number of states is defined by the equation
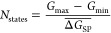
1where *G*_max_ and *G*_min_ are the maximum and minimum *G* values extracted from the full *G* swings, and  is the mean value of the conductance updates during the 1 pulse up–1 pulse down procedure. A graphical description of this function is depicted in Figure 3(a). To take into account cycle-to-cycle variability, we calculated the *G*_max_ and *G*_min_ values by averaging over 10 full *G* swings.

**Figure 3:**
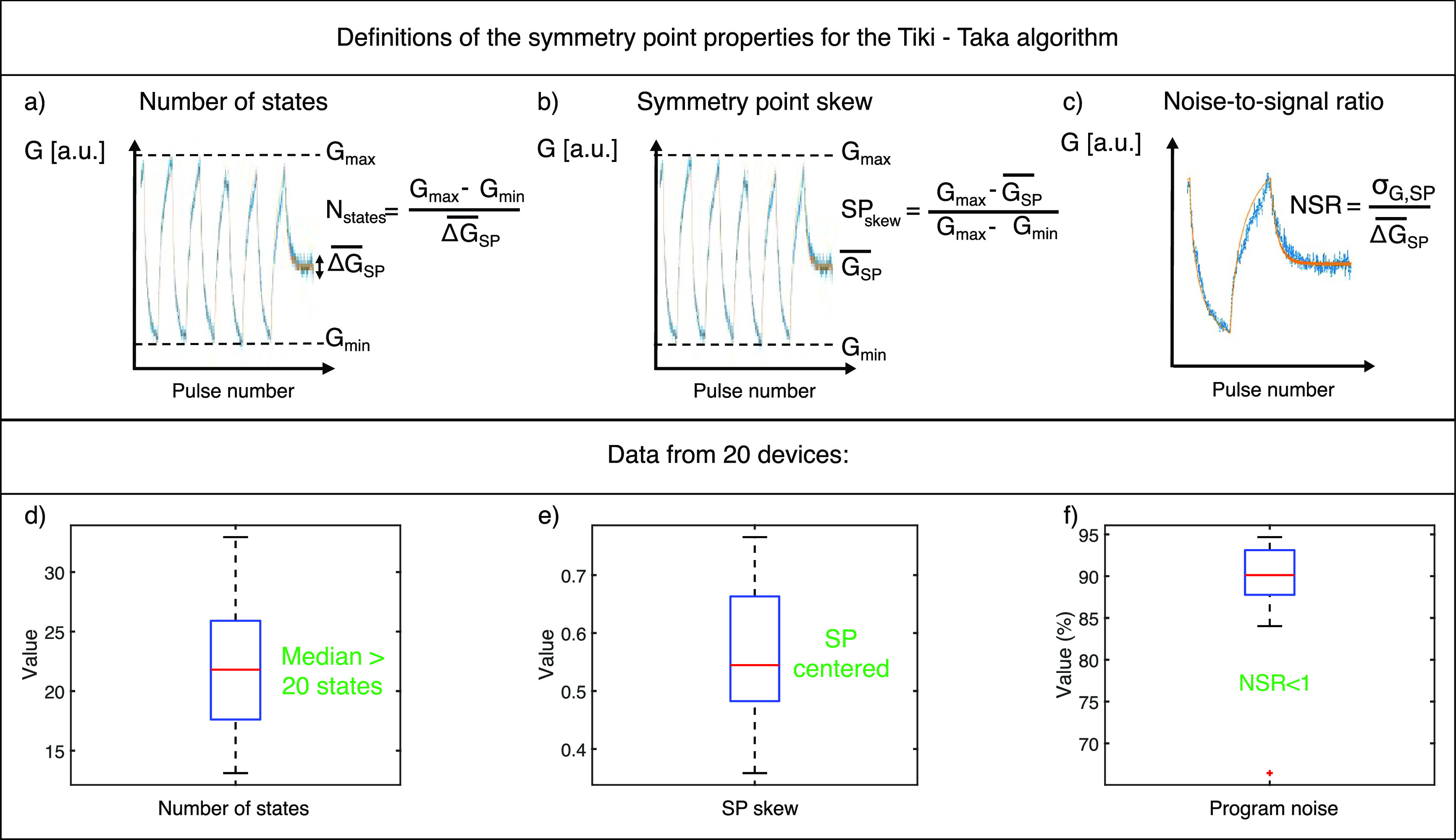
(a) Definition of the number of states, based on the presence of a symmetry point within the *G* swing. (b) Definition of the symmetry point skew. From this equation, the ideal symmetry point skew is 50%, which enables the maximum gradient accumulation in both directions. (c) Definition of the noise-to-signal ratio, as the capability to measure minimum changes of the device state. (d) Boxplot of the number of states measured from a sample of 20 devices. (e) Boxplot of the symmetry point skew. (f) Boxplot of the noise-to-signal ratio.

The SP skew is defined by the equation:
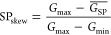
2where  is the average *G* state measured during the 1 pulse up–1 pulse down procedure. Based on this definition, the optimal SP skew is 50%, while higher or lower values indicate a drift toward *G*_min_ or *G*_max_, respectively. An illustration of this equation is represented in Figure 3(b).

The noise-to-signal ratio (NSR) is defined by the equation:
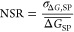
3where σ_*G*,SP_ is the standard deviation of the *G* updates during the 1 pulse up–1 pulse down procedure. An NSR < 1 is required for a successful execution of the Tiki-Taka algorithm, meaning the ability to discriminate between pulses up and down around the SP. A graphical explanation of this property is shown in Figure 3(c).

We determined the values of these functions for a set of 20 Gen_2_ devices. The results are reported as boxplots in Figure 3(d)–(f). The number of states ranges from a minimum of 13, to a maximum of 33, with a median value of 22 (see Figure 3(d)). The SP skew is contained between 36% and 77%, as displayed in Figure 3(e). With a median value of 54%, we notice a mild tendency to skew toward the *G*_min_ value. Figure 3(f) shows that the NSR is always lower than the acceptable maximum (<1). Generally, there is a trade-off between a low NSR and a high number of states, which is discussed in Supporting Information Figure S4.

Open source models of resistive switching devices are available on the “*aihwkit*”^30^ platform developed by IBM. In this work, we used the “*SoftBounds*” model from the *aihwkit* to fit the pulse response of our Gen_2_-RRAM devices. The fit is obtained by minimizing the average deviation of a parametric function to the experimental data traces, using Powell’s conjugate direction method^31^ (lmfit toolbox^32^). The results are displayed in Figure 4(a). The switching characteristics of 20 devices were used to take into account interdevice variability. Some examples are shown in Figure 4(b). To account for device-to-device variability, we fitted the conductance response model to the full traces of 20 different devices. With such a method, we enabled:

**Figure 4:**
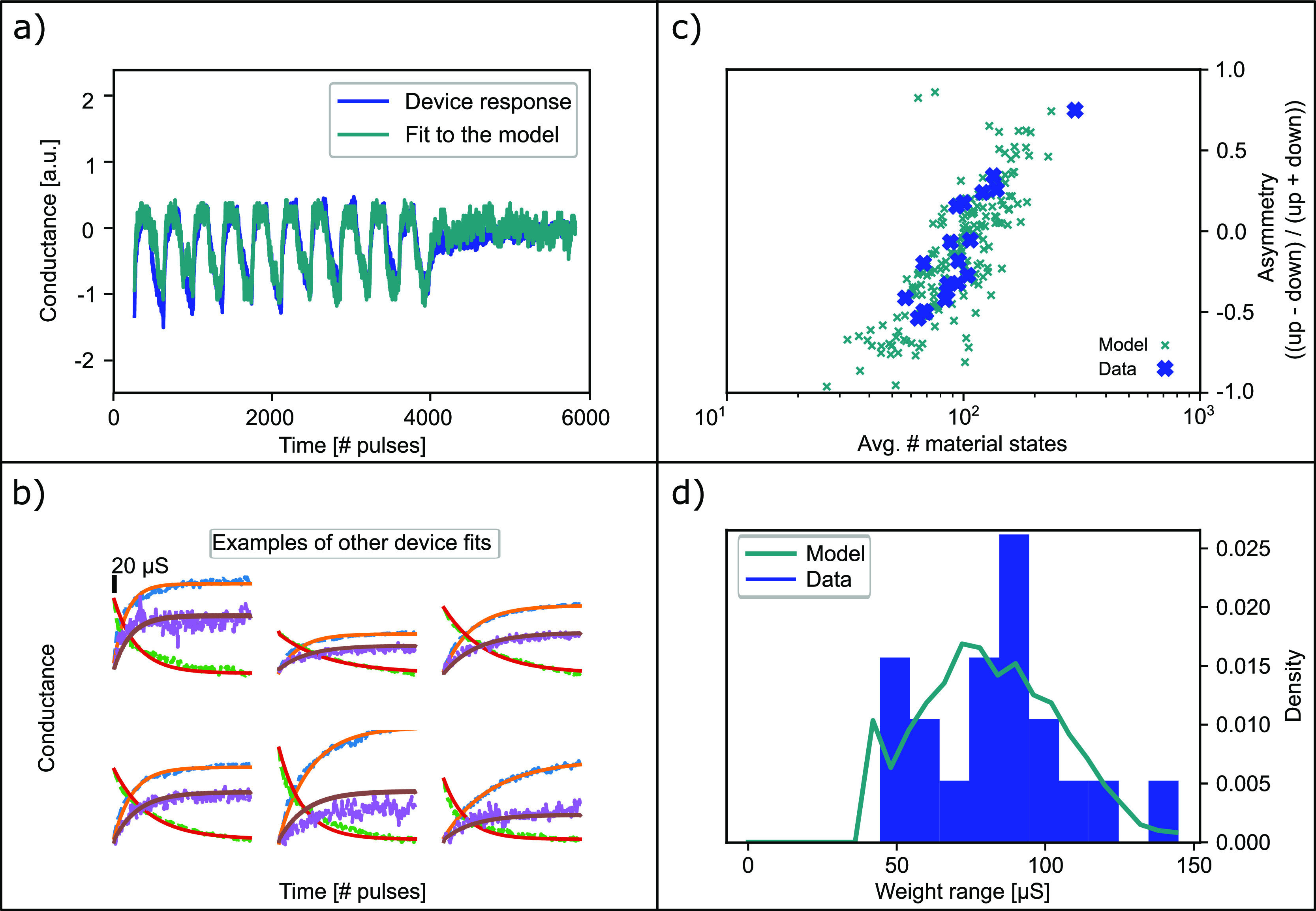
(a) “*SoftBounds*” model fitting the analogue response of a Gen_2_-RRAM device. (b) Collection of other device fits, which we used to include variability to the device switching model. (c) Pulse update asymmetry vs number of states, calculated from the device switching models. (d) Distribution of the *G* states calculated from the device switching models.

- Fitting the device-to-device variability in one variable independent of the others (e.g., *N*_states_ or *G*_max_);
- Fitting the correlated variation of multiple variables together, thus resulting in more realistic simulations compared to previous studies.

We analyzed these device fits, specifically the slopes of the curves, to recalculate the figure of merit number of states, as shown in Figure 4(c). Considering that the measured *G* window is given by 200 consecutive pulses in each direction, which can be insufficient for the saturation of the curves, the model returns parameters more reliable than those of the measurements.

Using the described device model, we simulate the training of a 3-layer FCN on the MNIST data set.^33^ In Figure 5(a), we compared the accuracy per epoch for the 32-bit floating point (FP) reference (in blue) and Tiki-Taka version 2 (*TTv2*) (in orange). After 100 training epochs, a drop of only 0.7% in accuracy is achieved with *TTv2* compared to the digital reference. The training accuracy achieved with our TaO_*x*_/HfO_*x*_ RRAM devices is 97.4%, higher compared to reference (21) (96.4%), where device variability was not modeled.

**Figure 5:**
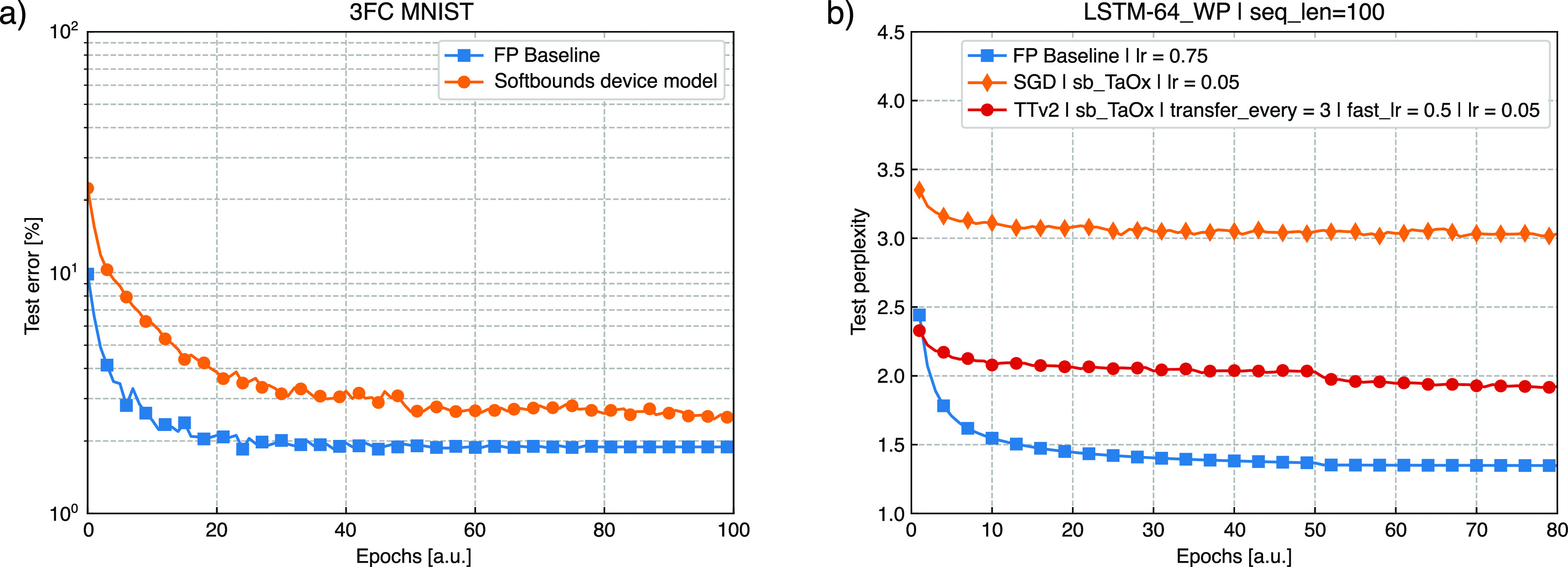
(a) Training simulation of a 3-layer FCN on the MNIST data set. Analog crossbars using *TTv2* (in orange) and a digital processing unit using 32-bit floating point (FP) resolution (in blue) are compared. (b) Training simulation of a 2-layer LSTM network, with hidden vector size = 64, for character prediction on the “*War and Peace*” novel (*LSTM2-64-WP*). The LSTM input sequence length is 100 characters (*‘seq_len’* = 100). We performed three training simulations. In the first one, we used digital synaptic weights (FP baseline, in blue) and a learning rate “*lr*” = 0.75. In the other two simulations, we used the analogue hardware model built from the “*SoftBounds*” fits of our TaO_*x*_ devices (*‘sb_taox’*). We compared the SGD algorithm (orange) to *TTv2* (in red). For the SGD algorithm, we updated the weight matrix with a learning rate *lr* = 0.05. For *TTv2*, we used a learning rate *‘fast_lr’* = 0.5 to update matrix (A), where gradient accumulation is performed.^23^ The forward pass of the weight matrix (C), the backward pass, and the update of matrix A are repeated every *‘transfer_every’* = 3 times. Then, a hot-encoded vector reads out matrix A and updates the hidden matrix (H)^23^ with a learning rate *lr* = 0.05.

We extend the simulation to a larger NN made of two stacked LSTM blocks with a hidden vector size of 64, followed by a fully connected layer. We used such NN for character prediction, based on the *War and Peace* novel as a data set. The results are shown in Figure 5(b), where the exponential of the cross-entropy loss, the test perplexity,^34^ is plotted over 80 training epochs. We observe that *TTv2* largely outscores the SGD algorithm by more than 30%, approaching the FP reference. This result shows that *TTv2* training with the estimated device properties generalizes to more complex NN workloads than MNIST.

In conclusion, we first presented how the main obstacle to realizing analogue AI accelerators based on resistive memory arrays is the nonideality of the device response to programming pulses. Then, we discussed how such a barrier can be overcome by co-optimizing algorithms and devices. In this manuscript, we showed:

- Improved training performances by using the Tiki-Taka algorithm, compared to SGD, on nonideal memristors.
- RRAM devices exhibiting enhanced linearity and symmetry, compared to baseline filamentary structures. Such improvements led to excellent symmetry point properties, which are the important device properties for the Tiki-Taka algorithm. Our RRAM structures combine more than 20 programmable states on average (more than 30 states as the best case), a centered symmetry point, and low programming noise. Moreover, excellent endurance (analogue properties preserved after more than 10^7^ programming pulses) and retention (drift of the analogue states <4% after baking at 85 °C for 72 h) were demonstrated.

We built a device model accounting for cycle-to-cycle and device-to-device variability. We showed that *TTv2* generalizes to more complex AI tasks, such as character prediction from the *War and Peace* novel, outperforming the more conventional SGD algorithm (training perplexity reduced by 30% compared to SGD).

## Supporting Information Available

Additional details on thin-film metrology, materials and electrical characterization, device modeling, and fabrication methods. This work is funded by the European Union within the H2020 “MANIC” (grant ID: 861153), “MeM-Scales” (grant ID: 871371), “NEoteRIC” (grant ID: 871330), “PHASTRAC” (grant ID: 101092096), “NEBULA” (grant ID: 871658), and “PlasmoniAC” (grant ID: 871391) projects and by the SNF “ALMOND” (grant ID: 198612) (PDF)
