# Supplementary material for: Analog Resistive Switching Devices for Training Deep Neural Networks with the Novel Tiki-Taka Algorithm
Source: Nano Lett. 2024 Jan 11;24(3):866–72. doi: 10.1021/acs.nanolett.3c03697 (PMC10811689; doi:10.1021/acs.nanolett.3c03697)
Supplement: Supplementary file 1 — nl3c03697_si_001.pdf [file nl3c03697_si_001.pdf]

Supporting information to manuscript:

"Analog resistive switching devices for training deep neural networks with the novel Tiki-Taka algorithm"

Tommaso Stecconi[1*], Valeria Bragaglia[1], Malte J. Rasch[2], Fabio Carta[2], Folkert Horst[1], Donato F. Falcone[1], Sofieke C. ten Kate[1], Nanbo Gong[2], Takashi Ando[2], Antonis Olziersky[1], and Bert Offrein[1]

[1] IBM Research Europe - Zürich, Rüschlikon, Zürich, CH 8803

[2] IBM Research - Yorktown Heights, Yorktown Heights, NY, US 10598

[*] E-mail: tec@zurich.ibm.com

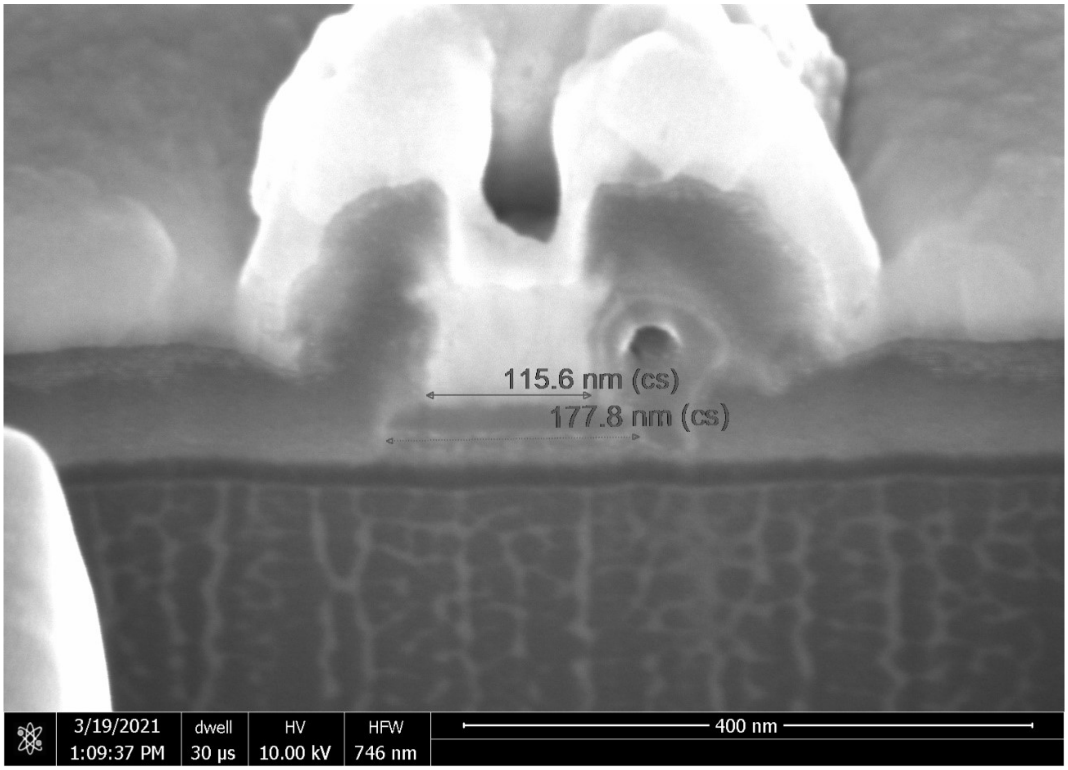

Figure S1: cross-section of a Gen_1-RRAM, with unit cell nominal area of $(200\ nm)^2$. Isotropic etching of the top layers causes uncontrolled definition of the device active area (the size of the TiN top electrode is measured to be only ~ 180 nm). Also, the sidewall concavity generates voids during the cladding of the active layers.

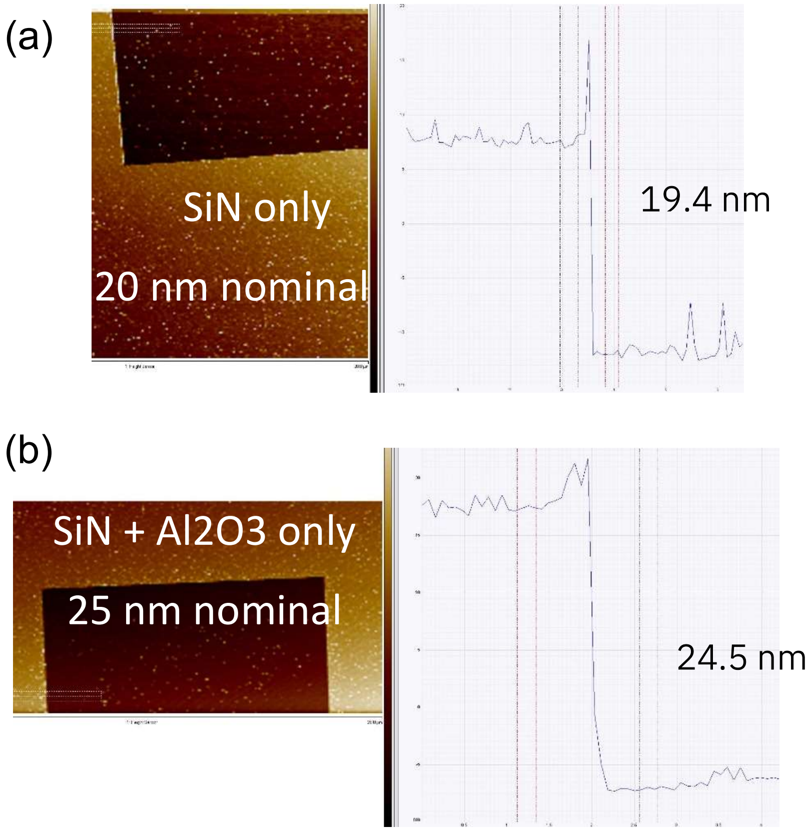

Figure S2: AFM scans before (a) and after (b) the Al2O3 removal by wet etch. We measure a step variation of 4.9 nm, corresponding to the 5 nm Al2O3 deposited by ALD. Therefore, the underlying HfOx layer is not attacked.

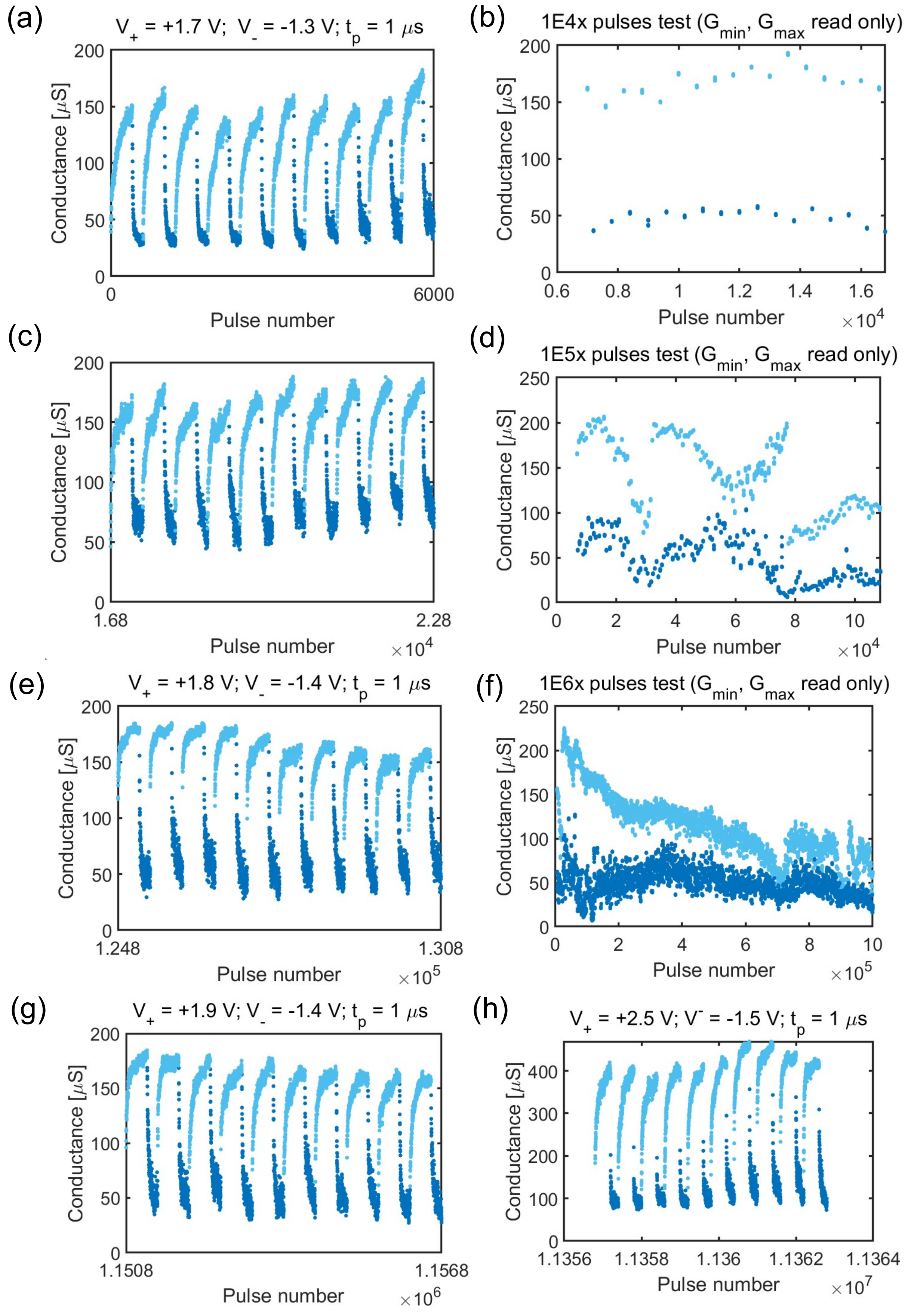

(a)
V+ = +1.7 V; V- = -1.3 V; tp = 1 μs
Conductance [μS]
Pulse number
(b)
1E4x pulses test (Gmin, Gmax read only)
Conductance [μS]
Pulse number
×10^4
(c)
Conductance [μS]
Pulse number
×10^4
(d)
1E5x pulses test (Gmin, Gmax read only)
Conductance [μS]
Pulse number
×10^4
(e)
V+ = +1.8 V; V- = -1.4 V; tp = 1 μs
Conductance [μS]
Pulse number
×10^5
(f)
1E6x pulses test (Gmin, Gmax read only)
Conductance [μS]
Pulse number
×10^5
(g)
V+ = +1.9 V; V- = -1.4 V; tp = 1 μs
Conductance [μS]
Pulse number
×10^6
(h)
V+ = +2.5 V; V- = -1.5 V; tp = 1 μs
Conductance [μS]
Pulse number
×10^7

Figure S3: (a) start of the endurance test and (b) next 1e4× pulses. (c) Check of the stability of the G window (after ~ 1e4× pulses). (d) Next 1e5× pulses. Random fluctuations of the G swing up to the first ~7E4× pulses, then the G swing reduces and skews towards lower G states. (e) To restore the initial G window after ~1e5× pulses, we increase the V+ and V- pulse amplitudes. (f) Next 1e6× pulses. Again, the G swing gradually reduces and skews towards lower G states. (g) To restore the initial G window after ~1e6× pulses, we increase the V+ and V- pulse amplitudes. (h) After ~1e7× pulses, we need to increase V+ up to 2.5 V and V- up to 1.5 V to achieve analogue synaptic potentiation and depression. However, the G window is now changed (Gmax is more than doubled).

(a)

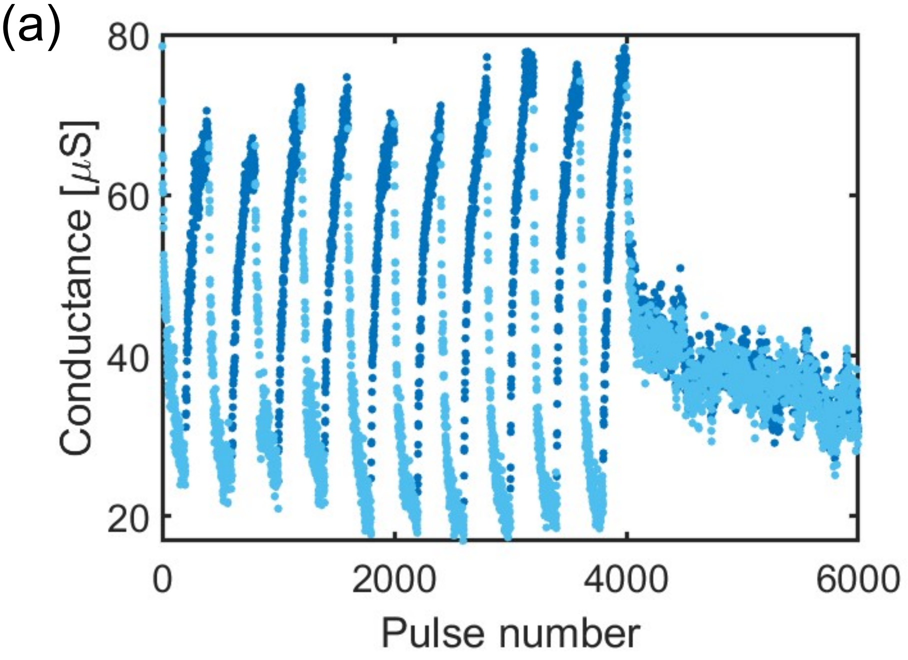

(b)

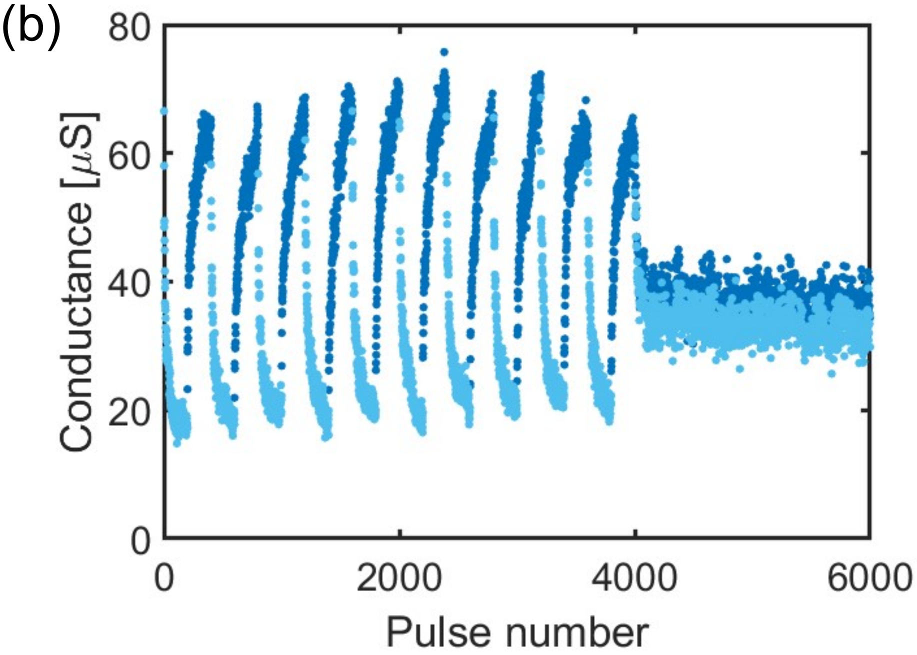

Figure S4: (a) Symmetry point with high *noise-to-signal (NSR)* ratio (93%). The pulses up and down can hardly be discriminated. However, since they're so small compared to the full G swing, the parameter *number of states* is high (26 states).

(b) Symmetry point with low *noise-to-signal (NSR)* ratio (66%). The pulses up and down can easily be discriminated. However, since they're large compared to the full G swing, the parameter *number of states* is low (13 states).

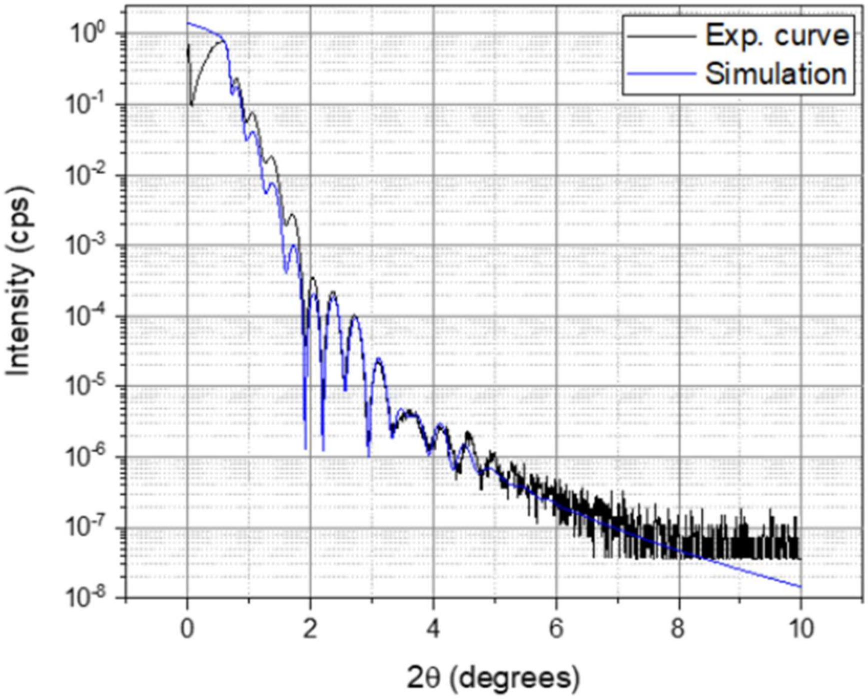

Material	Thickness [nm]	ρ [g/cm^3]	Roughness [nm]
$HfO_{x<2}$	3.5	8.4	0.8

Figure S5: XRR profile of the HfO_x layer used for Gen_2 devices and the table of the parameters used for fitting. The substrate is TiN (20 nm)/Si

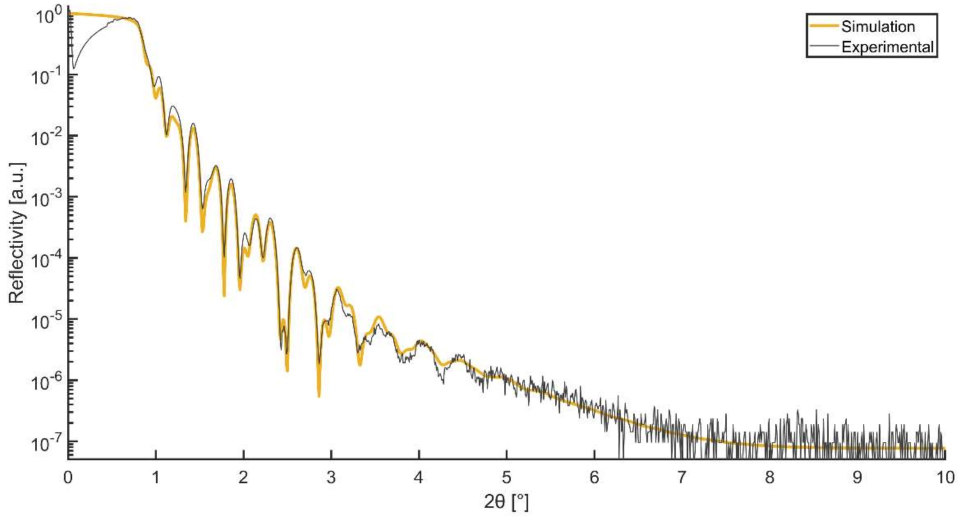

Material	Thickness [nm]	ρ [g/cm^3]	Roughness [nm]
$TaO_{x<2}$	26.4	10.7	1.4
Interfacial TaO_x	2.3	9.7	0.8

Figure S6: XRR profile of the TaO_x layer used for Gen_2 devices and the table of the parameters used for fitting. The substrate is SiO_2 (100 nm)/Si.

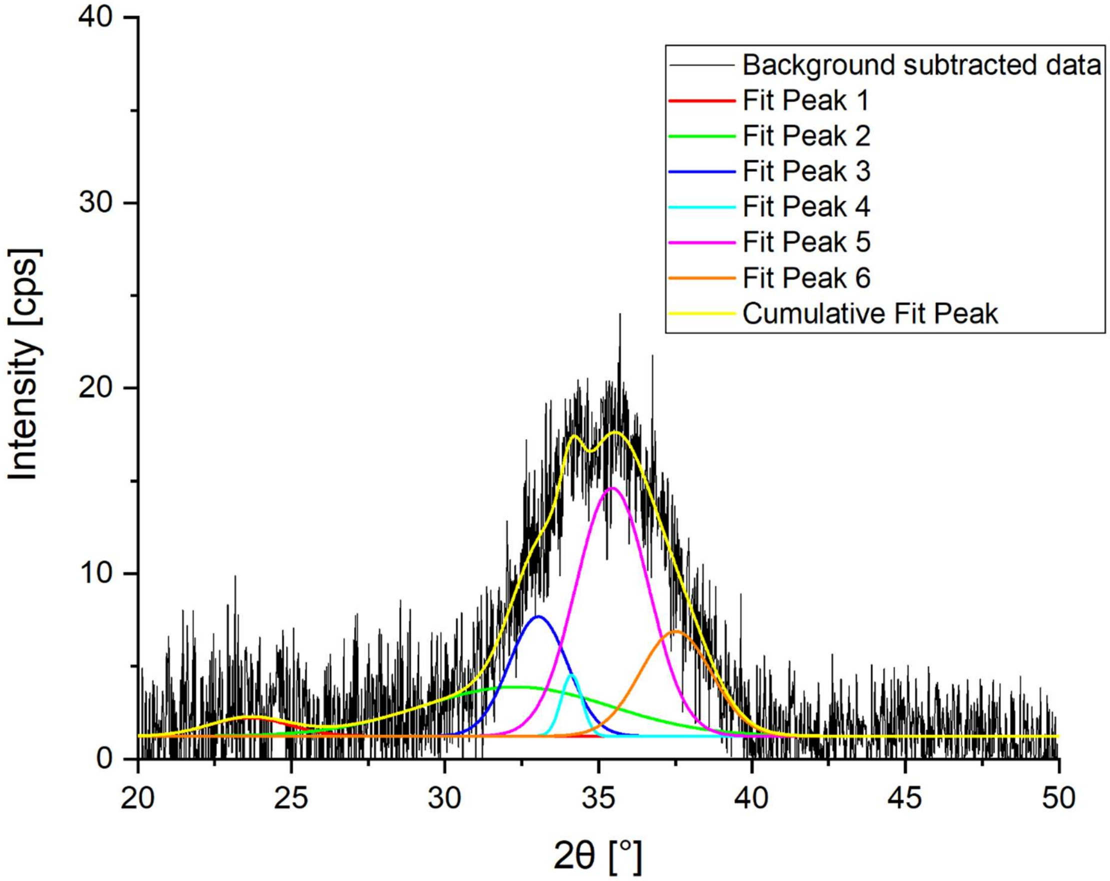

Figure S7: Grazing Incidence X-ray Diffraction (GIXRD) profiles of the TaO_x layer used for Gen_2 devices, with the peaks of the Gaussian deconvolution highlighted.

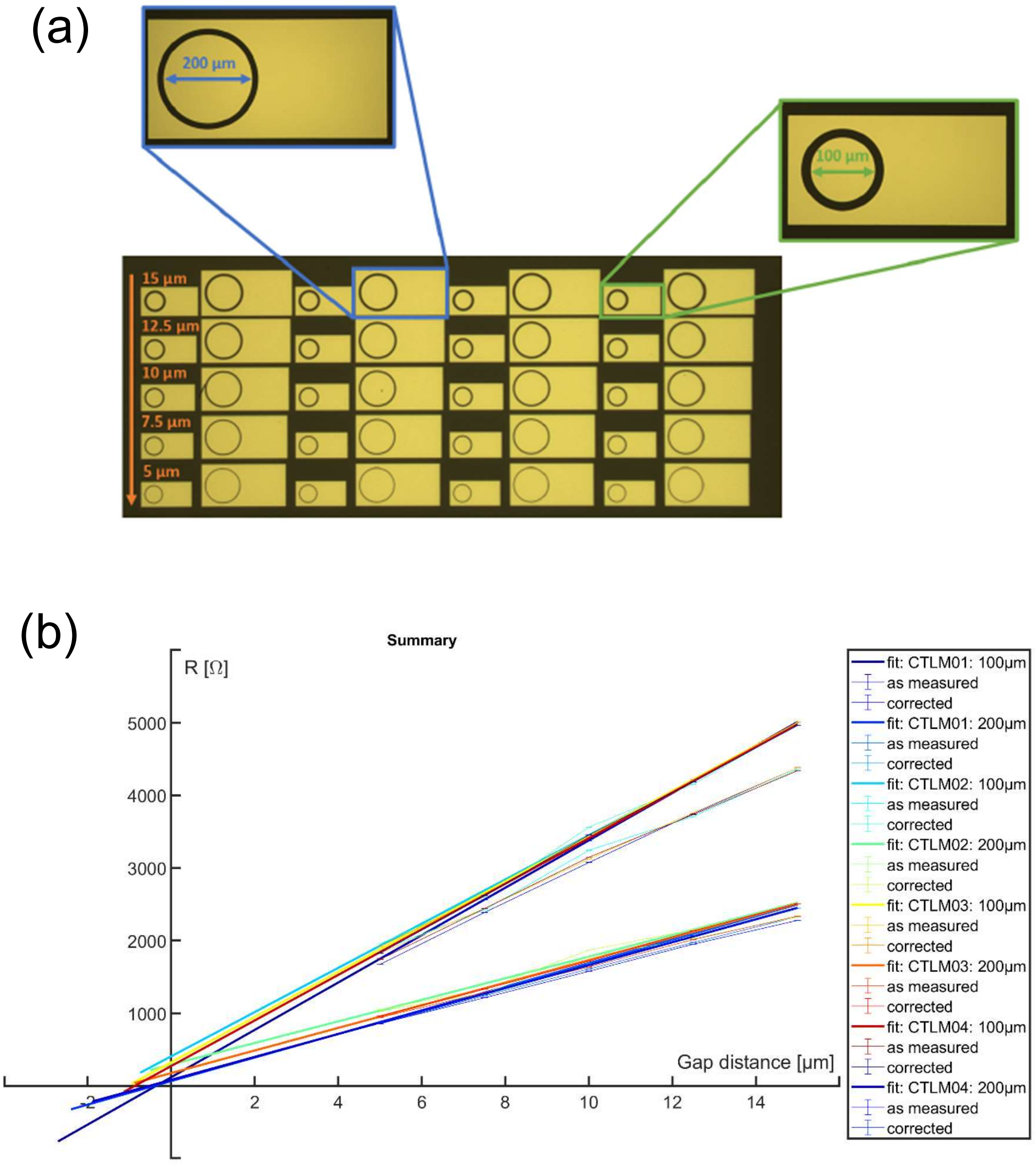

Figure S8: (a) Circular Transmission Line Measurements (C-TLM) structures used to calculate the sheet resistance of the TaOx material.

(b)Summary of the measurements, from which we extracted R_{sheet} = 98319.94 Ohm/square and resistivity = 0.281195.

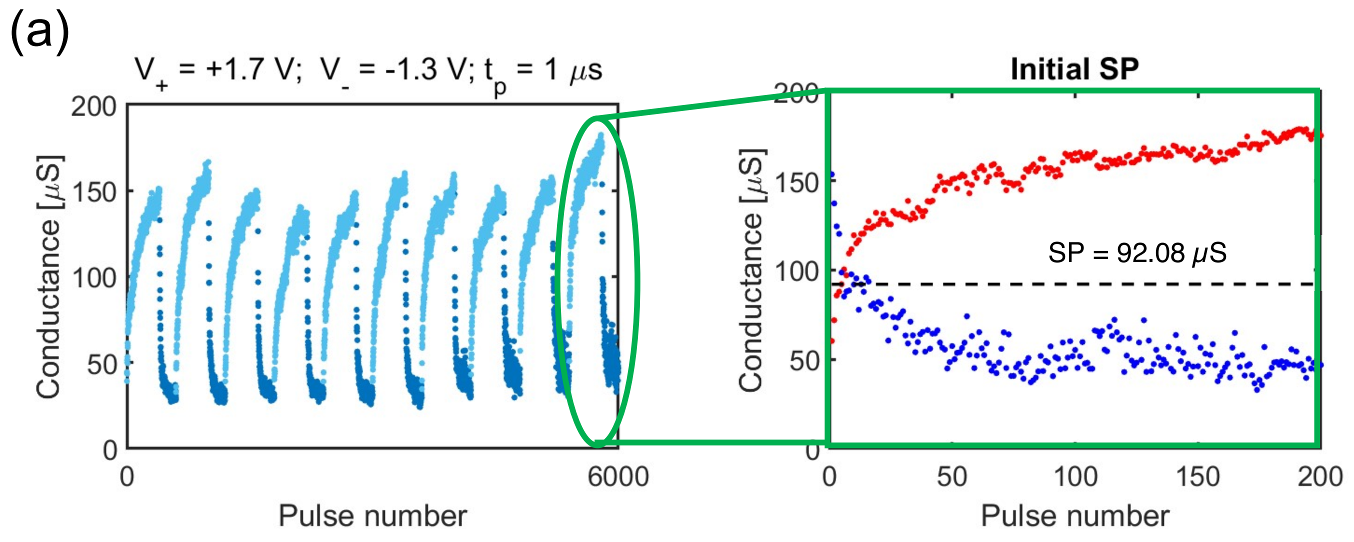

$$\frac{std(SP_{init})}{mean(SP_{init})} = 0.0858$$

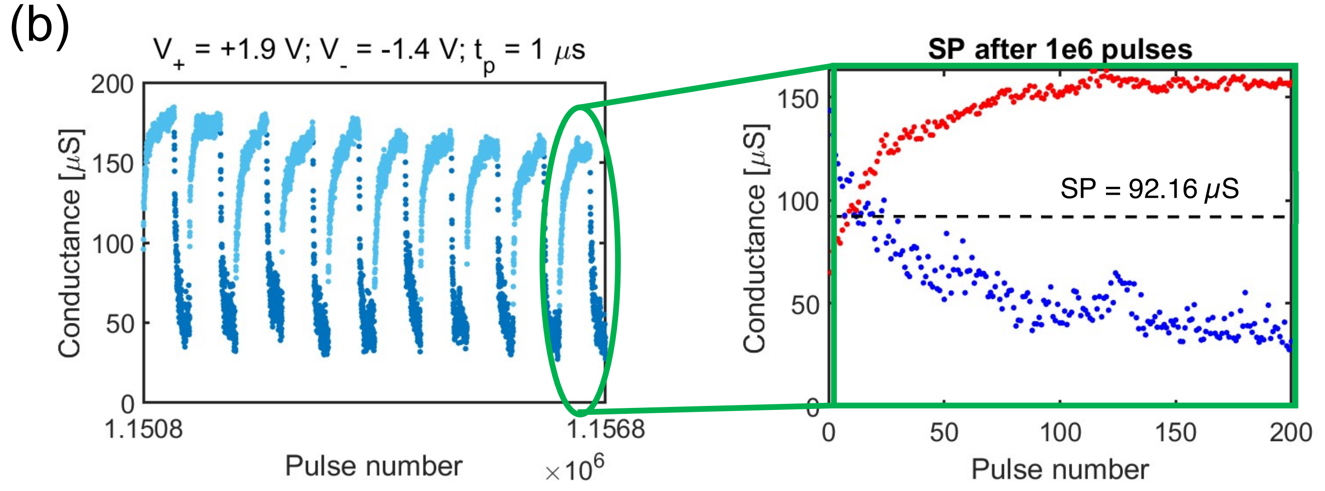

$$\frac{std(SP_{10^6})}{mean(SP_{10^6})} = 0.0880$$

Figure S9: (a) Symmetry Point (SP) analysis performed on the first 10x potentiation/depression cycles. The cycle-to-cycle variability of the symmetry point is computed as the ratio between its standard deviation and its mean value over multiple consecutive programming cycles. (b) Same analysis, performed after 1E6x pulses and V+, V- pulse amplitude correction.

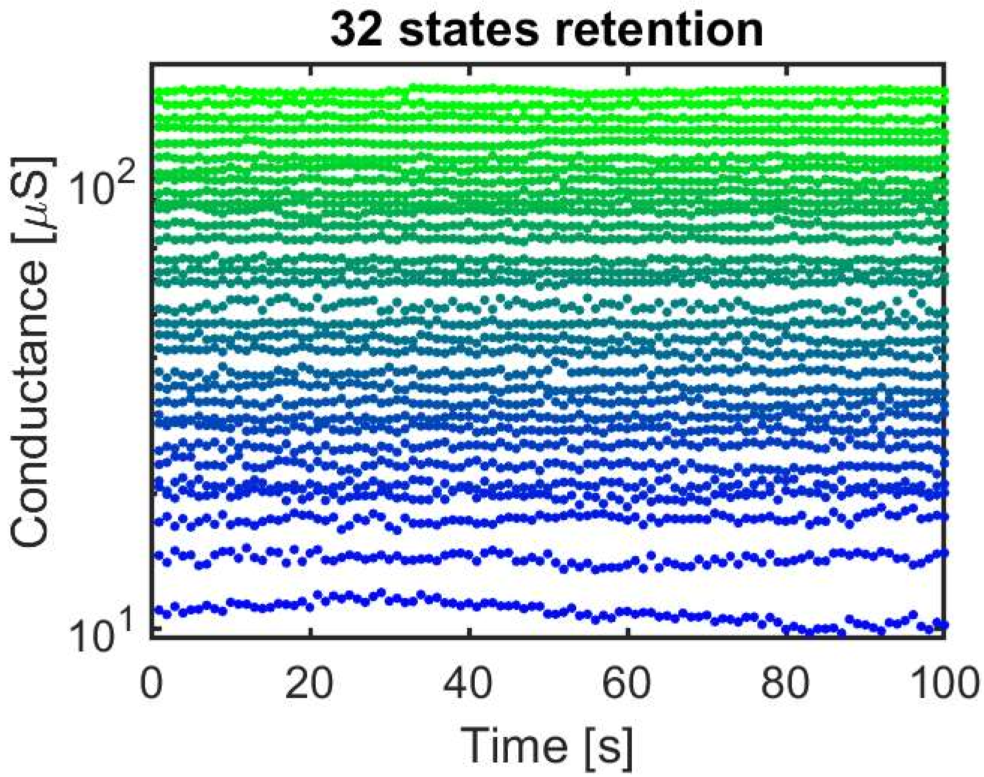

Figure S10: 32 states retention measurement.

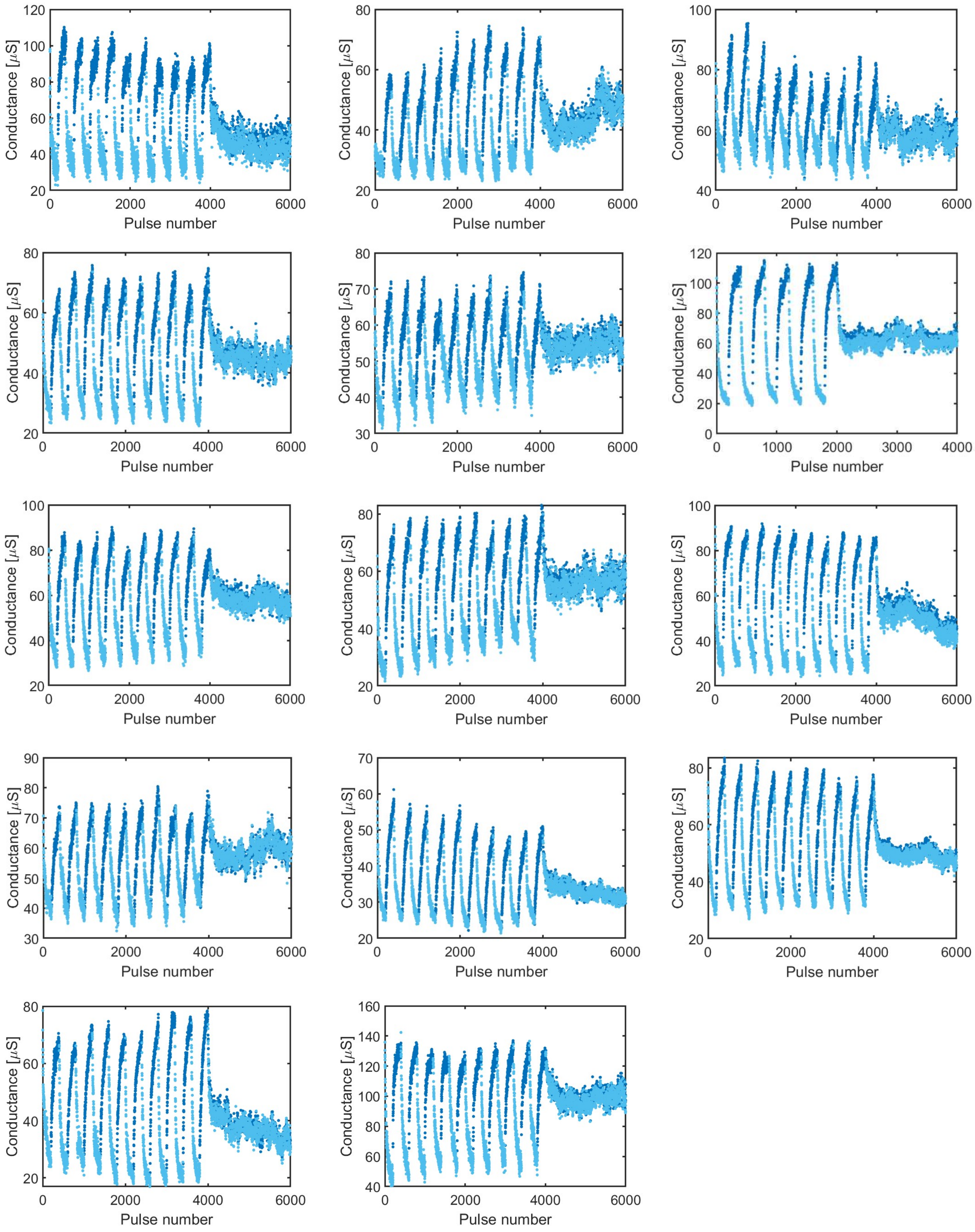

Conductance [μS]
Pulse number

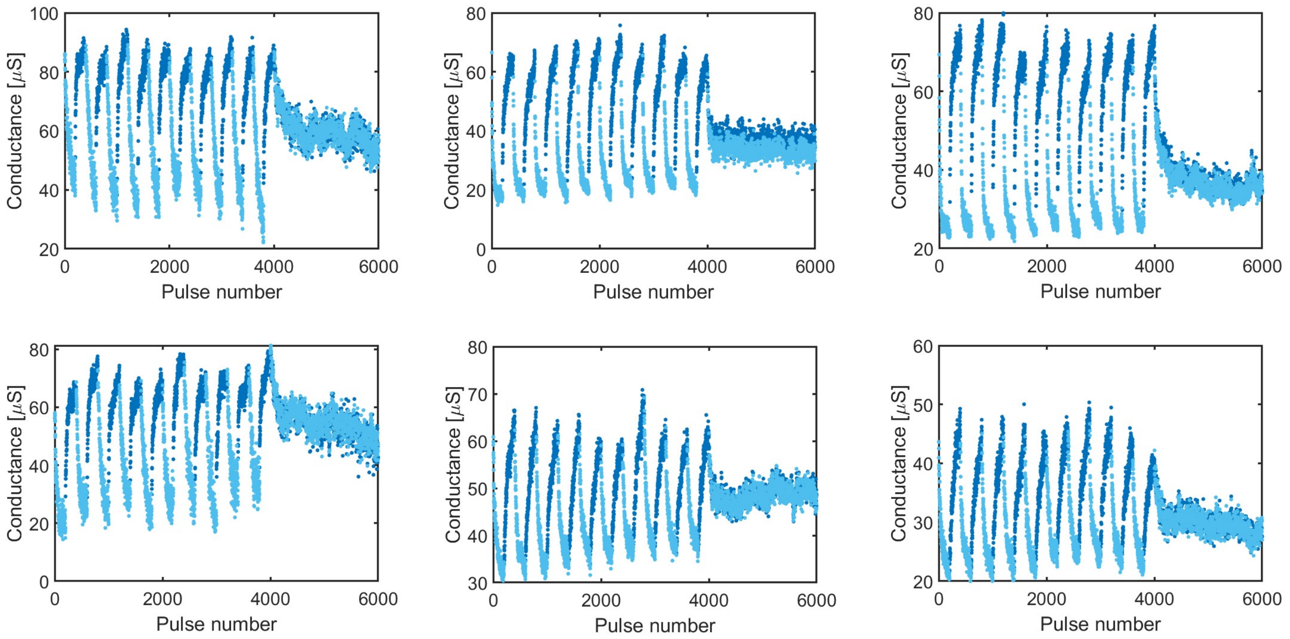

Figure S11: Variability data (potentiation + depression + symmetry point) extracted from 20 devices. Each figure shows a device response to 10x(200 pulses up + 200 pulses down) + 1000x (1 pulse up + 1 pulse down)

Methods

Fabrication

A sketch of the cross-section of the RRAM devices is depicted in Figure 1 (a) of the manuscript. A 20 nm thick TiN bottom electrode is deposited by plasma-enhanced atomic layer deposition (PE-ALD) at 300 °C, using a tetrakis-(dimethylamino)titanium (TDMAT) precursor and (N_2, H_2) plasma. The 3.5 nm thick HfO_x layer is deposited by PE-ALD at 290 °C using a tetrakis-(ethylmethylamino)hafnium (TEMAH) precursor and O_2 plasma. These layers are covered by 5 nm of Al_2O_3 and 20 nm of SiN, deposited again by PE-ALD, using trimethylaluminum (TMA) and Si as precursors at 300 °C and 400 °C, respectively.

The patterning of the device geometry is performed in two steps. First, the SiN layer is etched by Reactive-Ion Etching (RIE), using CHF_3 and O_2. This process stops at the Al_2O_3layer. Then, we immerge the chip in 'AZ 726 MIF' developer, to selectively etch the Al_2O_3. Next, a 30 nm thick TaO_x layer is deposited by reactive sputtering of a Ta target in mixed (Ar, O_2 plasma. A 20 nm thick TiN TE is deposited by RF sputtering of a TiN target in a mixed (Ar, N_2) plasma. A 50 nm W layer is sputtered on top. The sputtering of the W/TiN/TaO_x proceeds without vacuum breaking between the deposition of the different layers, to avoid uncontrolled oxidation at the reactive interfaces. To isolate the top contacts of different devices, we etch the W/TiN/TaO_x with inductively coupled plasma (ICP), using a mixed CHF_3 and SF_6 plasma, which stops at the HfO_2 layer. A passivation layer of 100 nm thick SiN_x is grown by plasma-enhanced chemical vapor deposition (PECVD). The via to access the device TE is etched with a mixed CHF_3 and O_2 plasma by RIE. 100 nm of W are sputtered and then RIE etched to define the device pads.
The described process flow avoids any lift-off steps, which could not be performed in foundries.

Electrical characterization

The electrical characterization is performed using a NI PXIe-5451 arbitrary waveform generator to source the generated pulses to the device TE, and an oscilloscope NI PXIe-5164 to read the current signal flowing through the device BE. The pulsed read scheme consists of alternating positive and negative pulses with amplitudes of +-200 mV and a duration of 10 µs, to cancel out any potential measurement offset.

Device modeling

1) We modeled the 20 device traces shown in our response to Question 4 using the "*SoftBounds*" weight update model, which is described in the reference [8] (see: Rasch, Malte J., et al. "Fast offset corrected in-memory training." *arXiv preprint arXiv:2303.04721* (2023)). We report here below the definition of the updates up and down (dw+ and dw-, respectively):

$$dw_+ = \alpha_+ \frac{b_{max} - w}{b_{max}} (1 + \sigma_{c2c}\xi)$$

$$dw_- = -\alpha_- \frac{b_{min} - w}{b_{min}} (1 + \sigma_{c2c}\xi)$$

Where $\alpha_\pm$ is an asymmetry linear correction, b_{max} (b_{min}) is the maximum (minimum) value of the synaptic weight, *w* is the value of the synaptic weight before the update, σ_{c2c} is the standard deviation from cycle-to-cycle variability, ξ is a Gaussian centered in 0 with standard deviation equal to 1 (normal distribution).Therefore, the *dw* updates are a function of multiple variables (such as α_+, α_-, b_{max}, b_{min}, etc.).

2) From the 20 device fits, we created two new variables (the vectors **x** and **y**), that are functions of (α_+, α_-, b_{max}, b_{min}, **...**), in two steps:
   - We defined a vector $\mathbf{N_{states,up}}$ as:

$$N_{states,up} = \frac{b_{max}}{\alpha_+ * dw_{min}}$$

   Similarly, we defined $\mathbf{N_{states,dn}}$ as:

$$N_{states,dn} = \frac{b_{min}}{\alpha_+ * dw_{min}}$$

   - We defined two vectors **x** and **y (see the figure 4 (c) in the manuscript, also reported below)** as:

$$\boldsymbol{x} = \log\left(\frac{N_{states,up} + N_{states,dn}}{2}\right)$$
$$\boldsymbol{y} = \frac{N_{states,up} - N_{states,dn}}{N_{states,up} + N_{states,dn}}$$

Therefore, **x** and **y** reflect the modeled number of states and asymmetry for the 20 devices. The log() function enables to model the number of states using a lognormal distribution.

3) Then, we approximate the values of the **x** and **y** vectors by creating a multivariate Gaussian distribution (a 2D Gaussian). Such distribution is obtained by computing the mean values of vectors **x** and **y**, and their covariance. **In the manuscript, when we write: "Fitting the correlated variation of multiple variables together", we refer to the covariance of x and y, where x and y are functions of multiple variables from the "*Softbounds*" model.**

   In the Figure 4 (c) of the revised manuscript we show in dark blue the 20 values of the vectors **x** and **y**, and in light blue many samples from their Gaussian models. The plot is also displayed report here below for your convenience:

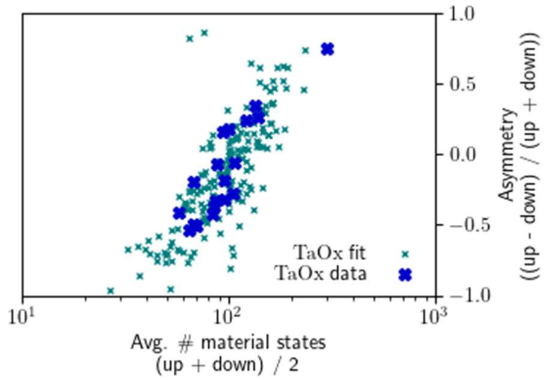

Figure 4 (c): Multivariate Gaussian fit (light blue) of the 20 device response models (in dark blue).
